# Thermoregulable Magnetic Microfluidic Devices by Magnetic Hyperthermia from Iron Oxide Nanoparticles

**DOI:** 10.1021/acsanm.5c01562

**Published:** 2025-07-11

**Authors:** Santiago Parames-Estevez, Pelayo García-Acevedo, Yago Radziunas-Salinas, Yolanda Piñeiro, José Rivas, Maria Teresa Flores-Arias, Alberto P. Munuzuri

**Affiliations:** † Group of Non-Linear Physics. Campus Sur. University of Santiago de Compostela, Santiago de Compostela, A Coruna 15705, Spain; ‡ Galician Center for Mathematical Research and Technology (CITMAga), Santiago de Compostela 15782, Spain; § Nanotechnology and Magnetism Lab—NANOMAG, Materials Institute−iMATUS, Health Research Institute−IDIS, Department of Applied Physics, 16780 Universidade de Santiago de Compostela., Santiago de Compostela E-15782, Spain; ∥ Photonics4Life Research Group, Applied Physics Department, Faculty of Physics and Materials Institute - iMATUS, Universidade de Santiago de Compostela, Campus Vida, Santiago de Compostela 15782, Spain

**Keywords:** organ on a chip, microfluidics, computer fluid dynamics (CFD), hyperthermia, magnetic nanoparticles, thermoregulable

## Abstract

Accurate control of energy supplied to a liquid in a controlled environment is essential for automating and optimizing processes. Organs on a chip (OoC) are an emerging technology that allows the design of customized environmental conditions for cells and chemical reactions by creating specific channel shapes while simplifying data acquisition. To thermoregulate these devices and therefore expand their use widely, we integrated iron oxide nanoparticles (IONPs) within the matrix of the chip to heat them by using magnetic hyperthermia. We tested the devices and developed a digital twin that reproduces the experimental OoC-fluid interaction while allowing us to measure parameters that would be inaccessible in a laboratory and get a full picture of the heat transfer at the boundary.

## Graphical abstract

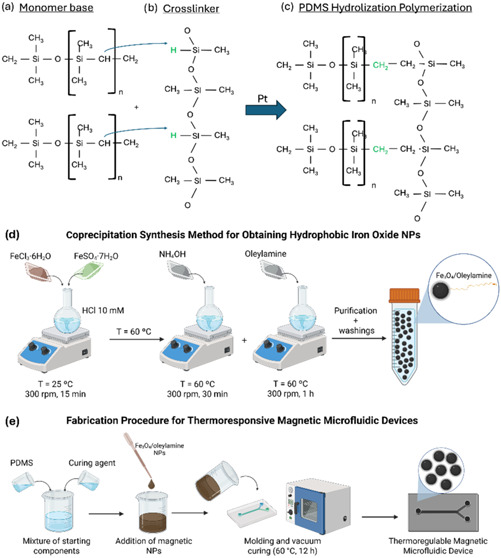

## 1 Introduction

Chemical processes often require complex and precise setups where compounds are sequentially mixed, treated, and transformed.^1^ This is particularly relevant for experiments with living cells where the process requirements are more delicate and can be solved by creating customized geometries.^2^ These examples have in common that their setups are usually constrained to the available standard instrumentation, making it costly or even impossible to design setups that optimize reactions or emulate the natural environments of cells. Organs on a chip (OoC)^3–5^ were created to satisfy this need, allowing the precise design of the geometrical conditions and minimizing the number of substances involved. This technology proves to be invaluable and has already been used in numerous applications, i.e., cells and complex structures tests,^6–8^ automatic reacting and nonreacting liquids mixing,^9–12^ and nanoparticle formation and characterization,^13,14^ to name a few.

Thus, it becomes apparent that the specific design of the OoCs strongly affects the outcome and functionality of the device. In fact, there are other physical properties that might also play critical roles in the process, in particular, the temperature. We propose in this work a method to construct a thermoregulated OoC device. The possibility of tuning the temperature in the boundaries of the chip and in the fluid flowing is a pivotal aspect for many applications in the fields of chemistry and medicine.

Regarding the fluid flow temperature, there are many catalytic reactions dependent on temperature gradients, such as the activation of enzymes^15,16^ or the phase transition in phospholipid membranes.^17–19^ Specifically, in the realm of soft matter physics, there are nanoparticle and microparticle systems whose crystallite size, morphology, or photoluminescence varies according to the temperature gradient of the liquid they are immersed in.^20–22^ Finally, it has been reported that temperature gradients can boost mixing due to autoconvection and thermodiffusion phenomena.^23–25^ This fact is a key parameter for the microfluidics realm, where mixing still is a challenge due to the minor Reynolds number, resulting in laminar flows.

On the other hand, controlling the temperature on the boundaries of the chip has many applications in the medical realm. There are many cellular processes dependent on temperature such as cellular migration. In their works, Khachaturyan et al.^26^ and Nakamura et al.^27^ showcased that leader and inflammatory cells are prone to migrate to regions in the body with a higher temperature in comparison with surrounding areas. This fact sheds light toward the comprehension of the wound healing processes in the body.

Nonetheless, the procedures followed to achieve the control of temperature rely on the setup of complex systems, where the samples are indirectly heated by means of heat transmission elements.^23^ These include thermosensors, Peltier elements, thermoresistors, contact heat transfer components, or quantum dots warmed by means of infrared lasers. The more elements are needed, the higher the complexity of the final device, thus making these procedures not as effective as wished.

In another approach, magnetic nanoparticles can be employed as a hyperthermia agent for oncologic purposes.^28–30^ Among the various types of magnetic nanoparticles, iron oxide-based nanoparticles are predominantly used due to their well-established biocompatibility, chemical stability, and favorable regulatory status, which have facilitated their clinical application. Although other magnetic materials such as cobalt or zinc ferrites can exhibit higher heating efficiency under specific conditions,^31^ concerns regarding their toxicity and instability in biological environments limit their widespread use. The heating mechanism employed, based on Néel and Brownian relaxation processes, is theoretically applicable to a broad range of magnetic nanoparticles; however, iron oxide nanoparticles remain the preferred choice for safe and effective hyperthermia treatment due to their proven safety profile.^32–34^

Nonetheless, there are currently limited works making use of magnetic nanoparticles within the microfluidics realm. In one of the most relevant works, Mamami et al.^35^ developed a glioblastoma tumor-on-a-chip model where hyperthermia was triggered by the administration of a nanoparticle magnetic solution in the channels of the chip. Cancer cells were lysed after 30 min of magnetic hyperthermia. Under these scenarios, difficulties can arise from the introduction of the nanoparticle solution within the chip, resulting in leakages, clogging, and so on. In addition, the system loses its transparency when introducing the solution within the channels, making the optical inspection of the device complex during the flow assay.

In our approach, we propose a novel hyperthermia microfluidic chip, integrating iron oxide magnetic nanoparticles with a polymeric shell (oleylamine) within the chip. The possibility of combining the biofunctionality of these nanoplatforms as well as their magnetic properties led us to create a microfluidic device whose matrix could have a tunable temperature by means of magnetic hyperthermia.

For that purpose, we embedded iron oxide nanoparticles (IONPs) in the composition of the device that can be activated by means of an external magnetic field to release heat into the system^36–40^ and provide an active control of the fluid temperature inside the device. IONPs were encapsulated by a selected coating shell of oleylamine, a surfactant widely used in metallic nanoparticle synthesis. This approach ensures to have a precise control on the size of the IONPs^41,42^ as well as to have an homogeneous integration within the main component of the OoC device, polydimethylsiloxane (PDMS), a broadly used material for biocompatible devices.^43–45^

Under this work, Sylgard 184 PDMS was employed. Its cross-linking process is depicted in Figure 1a–c, showcasing both components of the elastomer.^46^ On the one hand, the monomer base is composed of a sequence of [Si­(CH_3_)_2_O] units ending with vinyl groups. On the other hand, the cross-linker (curing agent) is composed of a sequence with siloxanes and silicones, including Si–H groups. A hydrolyzation reaction takes place when the vinyl group from the base interacts with the Si–H groups from the cross-linker. The reaction is fostered by means of a platinum catalyst. The final sample is then thermally cured, resulting in a 3-dimensional network, solidifying the PDMS.

**Figure 1:**
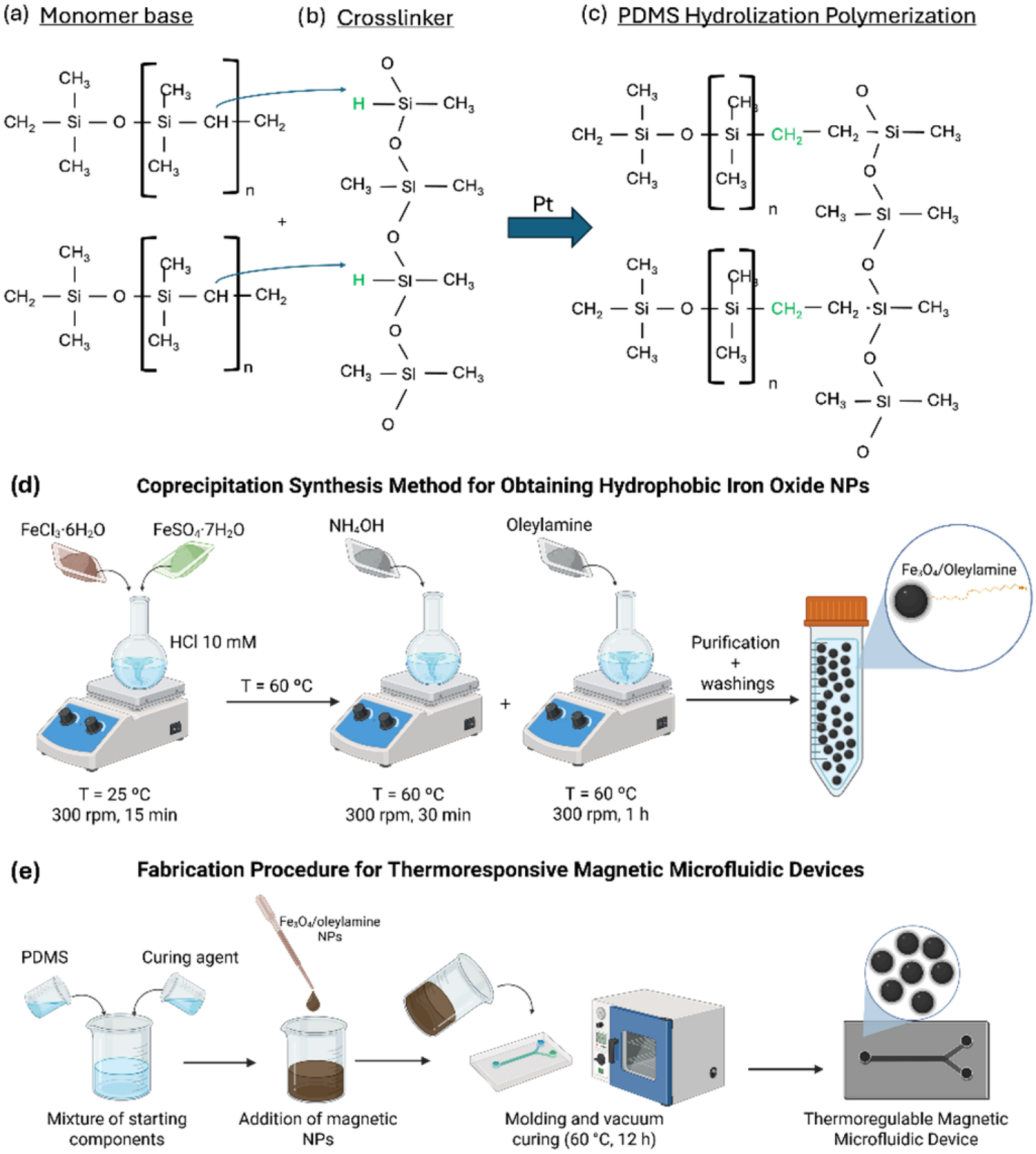
Scheme illustrates the chemical composition of PDMS and iron oxide nanoparticles. Regarding PDMS, the chemical structure of the two components is displayed, showcasing the (a) monomer base and the (b) curing agent. A hydrolyzation reaction takes place, resulting in a (c) 3D polymeric network. IONPs are synthesized by means of a (d) coprecipitation method, embedding them into the PDMS matrix after the (e) mixing process.

To the best of our knowledge, this is the first time that IONPs are integrated within the polymeric network, as seen in the coprecipitation procedures in Figure 1d–e, achieving a temperature-tunable device. It does not rely on complementary elements to transmit heat, thus making it simpler to work with. This approach could help us in further studies to create more complex chips with regions with and without IONPs. This would enable us to create interphases with thermal gradients, enabling us to mimic scenarios like thermal therapy in oncology and cardiology.

In addition to the benefits stated before, it is possible to take advantage of the metallic composition of the nanoparticles, as clusters can be located by using μCT (micro-CT). This characterization method is still under research in the medical realm, where gold, iodine, and bismuth nanoparticle systems are employed as contrast images.^47–49^ In the case of iron NPs, the technique is also employed for heavy metal accumulation analysis and magnetic particle inspection.^50^

Besides the integration of IONPs into PDMS, we went a step further in this work and studied how an internally circulating flux affects the thermal behavior of the microfluidic device. To fully understand and study the designed devices, we created a digital twin that accurately modeled the dynamics of the flows circulating inside and the temperature distribution within. The digital twin was accurately calibrated to provide detailed information.

The similarities in heat distribution in comparison of the experimental and theoretical approaches help us to completely characterize the chip. This work aims to build the first steps toward the development of a temperature gradient chip to be employed in both the chemical and medical realms by precisely controlling the temperature on the boundaries of the chip and the fluid flowing.

This work is organized as follows. First, we describe the method for fabricating a simple channel endowed with the thermoregulation system and show the properties of the different components. The following section is devoted to the characterization of the results obtained with the designed devices and the comparison with the digital twin.

## 2 Materials and Methods

### 2.1 Materials

Magnetic nanostructures were prepared by using the following chemicals without further purification. Iron­(II) sulfate heptahydrate (FeSO_4_·7H_2_O, 99%), Iron­(III) chloride hexahydrate (FeCl_3_·6H_2_O, 99%), ammonium hydroxide solution (NH_4_OH, 28%), and cyclohexane (C_6_H_12_, 99%) were obtained from Aldrich. Ethanol (C_2_H_6_O, absolute) was purchased from Merck. Hydrochloric acid (HCl, 37%) and oleylamine (C_18_H_37_N, C_18_ content 70%) were obtained from Acros Organics. Conversely, the microfluidic devices were produced using polydimethylsiloxane (PDMS) prepared from Sylgard 184 elastomer (Dow Chemical Company, Midland, Michigan). The material for the devices was made of Clear Resin V4 from Formlabs.

### 2.2 Synthesis of IONPs

The magnetic nanoparticles were prepared by using a coprecipitation method. FeCl_3_·6H_2_O (22 mmol) and FeSO_4_·7H_2_O (15 mmol) were dissolved in 50 mL HCl 10 mM with mechanical stirring at 300 rpm for 15 min. Then, the temperature was raised to 60 °C, and NH_4_OH (14 mmol) was added; the mixture was left to react for 30 min. Afterward, oleylamine (7.5 mmol) was added, and the mixture was left to react for 1 h. The mixture was then cooled down naturally to room temperature. The product was magnetically washed five times with a mix of water and ethanol 10:1. Finally, the nanoparticles were redispersed in cyclohexane.

The surface functionalization with oleylamine was strictly selected to ensure a hydrophobic nature of the IONPs, allowing their dispersion in organic media (cyclohexane). Given the inherently hydrophobic nature of PDMS—the main material used in the organ-on-a-chip platform—the use of a nonpolar solvent like cyclohexane is crucial to guarantee compatibility and achieve homogeneous incorporation of the IONPs within the PDMS matrix during device fabrication. Attempts to disperse nanoparticles in aqueous media resulted in poor integration and aggregation inside the PDMS, adversely affecting the functional properties of the device.

### 2.3 3D-Printed Mold Construction

First, the molds were conceptualized and designed with the help of a computer-assisted design (CAD) tool, Fusion360 (Autodesk, San Francisco, California). Following a previously optimized procedure,^51^ these molds are meticulously crafted with specific features intended to imprint onto the cast surface and sufficiently high walls to securely contain the cast material in its liquid state.

Subsequently, the finalized three-dimensional (3D) mold design undergoes a precision printing process that is made by using a stereolithographic 3D printer (Formlabs, Somerville, Massachusetts) and a commercial resin (Clear V4, Formlabs). The setup for printing this mask was chosen to be diagonal to enhance the structural integrity of the mold, ensuring robust and precise layering step-by-step. Following the completion of the printing process, the mold requires meticulous cleaning to remove any excess resin that could not be correctly evacuated during the polymerization process. A postcleaning process and final curing in a UV chamber were carried out. A final polishing of the mold surface is done to ensure adequate smoothness.

### 2.4 Magnetic Microfluidic Devices (MMD) Fabrication

First, nonmagnetic microfluidic devices were developed from a mixture of PDMS and a curing agent in a 10:1 ratio. The mixture was deposited in a mold previously fabricated by using the technique described in the section above, with the profile of the microfluidic channel. The mold is filled with this mixture and placed in a vacuum chamber for 40 min at 400 mbar to eliminate the bubbles produced in the mixing process. Finally, the device is cured in an LKN 86 furnace (Nannetti S.R.L., Faenza RA, Italia) at 60 °C for 12 h.

Magnetic microfluidic devices were produced by introducing magnetic nanoparticles during the microfluidic device fabrication process. For this purpose, 1 mL of the nanoparticles dispersed in cyclohexane at different concentrations (15, 30, 45, and 60 mg/mL) was added to the PDMS and curing agent mixture and integrated by stirring with a vortex. The mixture was then deposited in the mold, placed in a vacuum chamber for 40 min at 400 mbar to eliminate the presence of bubbles produced in the mixing process, and finally, cured in the furnace at 60 °C for 12 h. This vacuum and thermal curing process also ensured the complete evaporation of cyclohexane, preventing any residual solvent from remaining in the final device.

Five magnetic microfluidic devices (MMD) were produced with different amounts of nanoparticles: MMD_0_ (0 mg of nanoparticles), MMD_15_ (15 mg of nanoparticles), MMD_30_ (30 mg of nanoparticles), MMD_45_ (45 mg of nanoparticles), and MMD_60_ (60 mg of nanoparticles). After curation, the devices were bonded to a glass cover slide by activating the surface of the cover slide and PDMS with a Diener Zepto Plasma Cleaner and an oxygen atmosphere.

### 2.5 Flow Integration in Device Channels

To let the liquid in and out of the MMDs, two holes of 4 mm diameter were made at each extreme of the channel with a biopsy punch perpendicular to the slide. The extremal regions were designed to be wider to facilitate this operation and smooth the fluid movement at the ends. Silicone tubes were press-fit into the holes and connected to a peristaltic pump from Gilson, model Miniplus 3. The pump has four channels, each with an elastic silicone tube pressed against a wheel with cylindrical bearings; this results in a steady and controlled flux.^52^ To increase the speed, two channels were merged with a Y connection, leading to the input tube of the MMD. The liquid, water, was sourced from a beaker heated with a stove to emulate temperatures found in the human body. The device outlet also led to this beaker, closing the pneumatic circuit.

Three optical fiber thermometers were used to measure the critical temperatures in the system. Two of them were inserted into the circuit before and after the MMD with two T intersections, and the hole for the sensor insertion was plugged tightly with Parafilm to prevent leaks. The third sensor was placed in contact with the external surface of the device to monitor its temperature variation when a magnetic field was applied.

The flux of the peristaltic pump depends on how tight the tubes are pressed against the bearings. To know the influx (ϕ), the pump was calibrated by measuring the moved mass of liquid (*m*) in a time (*t*); the influx was $\phi =\frac{m}{t}$ . The measurement was repeated 5 times to estimate its uncertainty.

### 2.6 Characterization Methods

Different techniques were used to analyze relevant aspects of the components.

The characterization of the crystalline phase of the nanoparticles was performed by powder X-ray diffraction (XRD) using a Philips diffractometer (Panalytical, Callo End, UK) with Cu Kα radiation (λ = 1.5406 Å). Measurements were collected in the 2θ angle range from 10 to 80° with steps of 0.02° and an accounting time of 5 s per step. The peak broadening of XRD patterns is used to obtain crystallite size using Scherrer’s eq 1,

1 $$d=\frac{k\;\lambda }{\beta \;\cos\theta_{\mathrm{hkl}}}$$

where *d* is the mean size of the crystallite (nm), *k* is the Scherrer constant (0.9, dimension-less), λ is the wavelength of the X-ray beam used (0.154060 nm), β is the full width at half-maximum (fwhm, radians) of the peak, and θ is the Bragg angle (degrees).

The morphology of the IONPs was characterized by transmission electron microscopy (TEM) using a JEOL JEM-1011 microscope (JEOL, Tokyo, Japan) operating at 100 kV. This technique was selected due to its high resolution, allowing direct visualization and accurate measurement of nanoparticles at the nanometric scale. From these images, the size distribution of the IONPs was determined using ImageJ software, fitting the values to a distribution fit using OriginPro 2016 software.

The Fourier transform infrared (FT-IR) spectra were recorded in a Thermo Nicolet Nexus spectrometer (Thermo Fisher Scientific, Madrid, Spain) using the attenuated total reflectance (ATR) method in the range 400–4000 cm^–1^.

DC magnetization curves of dried samples were measured by using a Quantum Design SQUID magnetometer (Quantum Design, Darmstadt, Germany). The hysteresis loops were measured with an applied magnetic field between −25 and 25 kOe at 300 K.

Magnetic hyperthermia response of MMDs and liquid temperatures were measured using a commercially available setup (MagneTherm, Nanotherics) equipped with three optic fibers.

Transmittance at different wavelengths from NIR to UV was analyzed with a PerkinElmer Lambda 25 instrument to check if bright-field microscopy was still an option after adding nanoparticles to the device, making it opaque. Confocal images of the microchannels manufactured and superficial roughness measurements of the MMDs were obtained using a 3D optical profilometer S neox (Sensofar Metrology, Terrassa, Spain).

The final devices with and without nanoparticles were also evaluated by microcomputed tomography (μCT or micro-CT) using a SkyScan 1272 X-ray micro-CT instrument (Bruker; Kontich, Belgium). The obtained projections were reconstructed using Nrecon software (Bruker; Kontich, Belgium). The reconstructed images were volume-recorded using CTVox software (Bruker) to visualize the distribution of the NNPs in the volume of the devices fabricated in PDMS.

Finally, the surfaces of these devices were analyzed with scanning electron microscopy (SEM) using a GeminiSEM 500 (ZEISS) operating at 20 kV.

### 2.7 Hypothesis for the Numerical Model

In a magnetic hyperthermia assay, an external magnetic field applied to IONPs may induce two relaxation processes (Néel and Brown relaxations) that provoke heating of the particles. These two main mechanisms of energy dissipation, friction with the surrounding material because of the induced movement (Brown relaxation) and heating due to a change in the orientation of their magnetic moments (Néel relaxation), contribute differently when the IONPs are dispersed in a liquid (free to diffuse and rotate) or embedded in a solid matrix (limited degrees of freedom). Since, in this work, the IONPs were integrated into a PDMS structure, we hypothesize that the 3d polymeric network of the PDMS traps the IONPs, significantly reducing the heat produced by induced movement (Brown relaxation).

To create a digital twin that simulates the interaction between the external magnetic field and the MMD, the contribution of each energy dissipation path and its interaction with the alternating magnetic field must be precisely determined. Tremendous computational resources and time would be needed to simulate a significant amount of time (∼10 min), making this approach very inefficient. To overcome this difficulty, our approach seeks to replace the magnetic interaction by modeling the MMD as a heating element. This strategy requires, therefore, the estimation of the heating power of the device, which is addressed by measuring the temperature of the MMDs over time. The energy needed to change the temperature of the device has been approximated as Δ*Q* = mc_p_Δ*T*, where Δ*Q* is the change in energy, m is the device’s mass, *c*_p_ is the specific heat of the mix of NPs and PDMS, and Δ*T* shows how much the device’s temperature is changed. Using as a reference the temperature at equilibrium, Δ*Q* = mc_p_Δ*T* becomes

2 $$Q\left(t\right)-Q\left(t=0\right)=mc_{p}\left[T\left(t\right)-T\left(t=0\right)\right]$$

where *Q*(*t*) is the thermal energy that the device has at a given time, *t*, and *T*(*t*) is its temperature at the same instant, which can be modeled by deriving the transient heat equation^53^ as,

3 $$T\left(t\right)=T_{f}+\left(T_{0}-T_{f}\right)e^{-t/\tau }$$

where *T*_f_ stands as the device’s temperature at the new equilibrium, *T*_0_, its temperature at the old equilibrium (*T*(*t* = 0)), and τ, a characteristic time that determines how fast the process evolves.

Our hypothesis considers that all of the energy leaving the IONPs (due to Néel relaxation phenomena, primarily) is exclusively invested in heating the MMD immediately after turning the magnetic field on. Heat coming from the IONPs is released outward from the polymeric matrix (air surrounding, fluid flow, tubing, etc.) until equilibrium is reached. Once there, the temperature of the system stabilizes.

Under this situation, according to the concentration of the IONPs embedded in the device as well as with the intensity of the magnetic field, different temperatures can be obtained by means of hyperthermia. This allows us to access a tunable microfluidic system whose final temperature can be selected according to the application. As the particles are embedded in the PDMS matrix, conventional protocols for cell culturing can be applied. Once the cells are cultured, the device could be heated to approach temperatures at which the cellular fate can be studied. These studies are relevant in fields such as oncology, where it has been proven that heat affects most of the hallways of cancer,^54^ showcasing the effectiveness of hyperthermia combined with other therapies to treat cancer. We see this proof of concept as a first step toward the development of more complex OoC technologies with hybrid regions to study cellular activity according to the effect of different temperatures, all of them within the same device.

To deeply understand the mechanisms of heating in the device, we simulate its behavior by means of the digital twin. As stated before, the power of the device due to the IONP magnetic hyperthermia is required. In order to achieve it, we do assume that this power can be approximated as the variation of heat accumulation the first time the magnetic field is on, as depicted in eq 4.

4 $$P_{d}\approx \lim_{t\to 0^{+}\;}\;\mathrm{Q̇}\left(t\right)=\lim_{t\to 0^{+}\;}\;{\mathrm{mc}}_{p}Ṫ\left(t\right)={\mathrm{mc}}_{p}\frac{T_{f}-T_{0}}{\tau }$$

where the heating power of the device is *P*_d_ and *Q̇*(*t*) and *Ṫ*(*t*) are the time derivatives of the heating and temperature curves, respectively.

Therefore, to obtain the power, we recorded how the system heats over time. Since we know it behaves according to eq 3, we use the recorded experimental data to fit the heating curve. Using all of the curves (instead of a single measurement at *t* = 0 s) allows us to infer with more precision the slope of the said curve at *t* = 0 s, which approximates closely to the power that the device generates because exactly at *t* = 0 s, there is no temperature gradient with the environment, and all of the heat is invested in raising the temperature of the device.

This assumption allows us to characterize the device by measuring only its heating curve and its composition (mass and *c*_p_). The remaining parameters τ, *T*_0_, and *T*_f_ can be extracted by fitting heating curves to eq 3, and with this procedure, simulation times can be reduced to less than one simulated second per real second. The properties used to simulate the different materials involved can be seen in Table 1.

**Table 1:** Relevant Parameters of the Materials Involved in the Process^a^ ^55^

	ρ (kg m^–3^)	*c*_p_ (J kg^–1^ K^–1^)	κ (W m^–1^ K^–1^)
glass slide	2500	840	1.00
silicone	150	818	2.55
water	998	4184	0.62
PDMS	965	1460	0.15
iron	7874	440	80.2
device	965	1460	0.15

a The Parameters Are Density, Specific Heat, and Conductivity, Presented in the Table as ρ, *c*_p_, and κ, Respectively. All Material Properties Were Provided by the Material Database Integrated in Star-CCM+ except for PDMS.^55^

We tested our hypothesis by comparing the simulation with the experimental behavior of MMD_60_; the results will be explained in Section 3.5. Another piece of information that supports our hypothesis can be found in the data represented in Figure S1 of the Supporting Information (SI), where the experimental data for each ϕ have been fitted and extrapolated until all of the curves are at equilibrium. In Figure S1a, all cases start with very close slopes and diverge in different ways, dictated by their dissipation paths. In Figure S1b, the heat increment of the MMD slowly decreases as the device approaches equilibrium, transferring all of the heat produced to its dissipation paths (water, air, slide, etc.). In the adiabatic case, all of the produced energy would remain inside the device, increasing its temperature at a constant rate.

### 2.8 Digital Twin Design

To create the digital twin, all of the system’s components were modeled as separate objects in Fusion360 and simulated with their corresponding physical properties in Star-CCM+ (Siemens Digital Industries Software, Texas). The properties of the device shown in Table 1 are assumed to be those of PDMS, given the complexity to precisely determine the properties of the mixture, which is approximately homogeneous but still presents some imperfections, as shown by the SEM scan in Figure S2 and micro-CT images in Figure S8 (in the Supporting Information), where small clusters of IONPs can be seen at the surface and the whole volume as bright spots, due to their conductive nature. In Figure S3, the SEM scan of a control device’s surface with no IONPs is also shown to illustrate the aspect of a device made only of PDMS. Since the clusters of IONPs appear isolated, we simulated the device with properties of PDMS (thermal conductivity, specific heat, and density) since, in most of the volume, the heat and temperature will be transmitted through pure PDMS.

To reproduce the interaction of the device with its environment, additional parameters were included, such as the ambient temperature for air at the time of the experiments (26 °C), initial temperatures for MMD and water (36 °C), and the flux inside the MMD’s channel. This gives us all of the information needed to feed our digital twin. In the next section, we will discuss the properties of the used IONPs, the measured characteristics for our channels, a comparison between experimental and simulated temperatures at the end of the channel, and, finally, the ability of our digital twin to measure properties and variables that would otherwise be very impractical or even impossible.

## 3 Results and Discussion

### 3.1 Morphological and Magnetic Characterization of IONPs

The main physicochemical properties of the IONPs were analyzed by using relevant techniques in the field. The morphology and size were determined by TEM, as can be observed in Figure 2a. These images show that almost all IONPs present a quasi-spherical morphology with an average diameter of around (19 ± 4) nm. The crystalline structure was analyzed in terms of their XRD pattern. In the Supporting Information, Figure S4a shows their diffraction pattern and magnetite theoretical diffraction peaks (ICSD card No. 158742).^56^ It can be observed that the position and relative intensity of the IONPs’ diffraction peaks (obtained at 18.4, 30.2, 35.5, 37.2, 43.2, 53.6, 57.1, 62.7, 71.2, and 74.2°) match the primary theoretical magnetite peaks ([111], [022], [113], [222], [400], [224], [115], [044], [026], and [335] corresponding to reflections from planes of the Fe_3_O_4_ cubic lattice). The broadening of the peaks is expected to happen for small NPs^57^ and allows us to obtain the crystalline size from eq 1. In the present case, IONPs have a crystallite size of about (15 ± 3) nm, in concordance with the TEM-measured NP diameter. The FT-IR spectrum shown in Figure S4b allows us to confirm the presence of a specific coating shell over the magnetic cores. An absorption band can be observed at 543 cm^–1^, characteristic of the stretching vibrations of the Fe–O bond, indicating the presence of Fe_3_O_4_. The two bands around 2853 and 2924 cm^–1^ are assigned to the symmetric and asymmetric stretching vibrations of the CH_2_ groups, respectively. A final broad absorption band around 3300 cm^–1^ corresponds to the stretching vibrations of the N–H bond.^57^ These absorption bands confirm the presence of the polymer coating over the magnetite cores. The DC magnetic properties of the IONPs were analyzed by using magnetization cycles obtained from a superconducting quantum interference device (SQUID) magnetometer. The measurements were conducted within a field range of −25 to +25 kOe at a temperature of 300 K. Figure 2b shows the hysteresis loops of the IONPs from which relevant magnetic parameters such as saturation magnetization, MS = 75.50 emu g^–1^, remanence, MR = 0.26 emu g^–1^, and coercivity, HC = 2.28 Oe were obtained. Magnetization values, M, were normalized to the magnetic mass present in the sample, which was determined by thermogravimetric analysis, which, for the IONPs, accounted for 92% of magnetite. MR and HC values close to zero suggest the superparamagnetic behavior of the iron oxide nanoparticles, which is crucial to ensure their absence of magnetic interaction between the MNPs.

**Figure 2:**
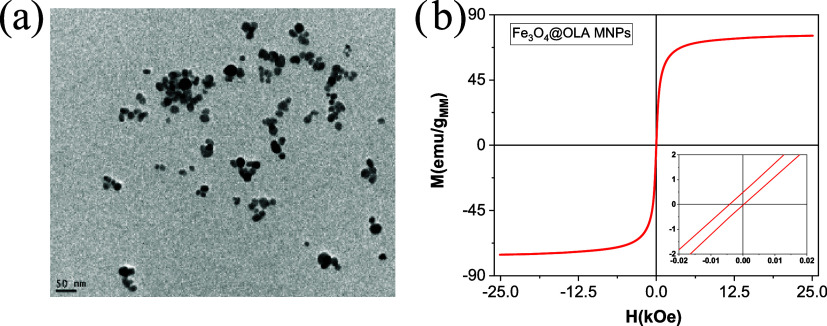
(a) TEM micrograph of IONPs. The size distribution was performed using ImageJ software, showing particle sizes of (19 ± 4) nm. (b) Hysteresis loops of IONPs at 300 K. Insets: scale amplification is observed in hysteresis loops. The hysteresis loops were normalized to the mass of the magnetic material (iron oxide) in the total assembly.

### 3.2 Design Parameters of Magnetic Microfluidic Devices

When designing the geometry of the magnetic microfluidic devices, we strived to create a platform on which we could introduce liquids circulating at arbitrary speeds while thermally regulating the temperature of the volume surrounding the flowing fluid. While this concept already has several possible applications involving microfluidic systems for continuous nonabrasive chemical reactions, it was of interest to explore solutions that would allow us to observe and study organic cells in future studies while keeping the samples in a portable format.

To create the magnetic microfluidic devices (MMDs), we first designed a mold in which we poured the PDMS doped with nanoparticles and cured it by following the steps already described in Section 2.4. The mold was designed as a square recipient with sides of 2.4 cm and a height of 1 cm. At their base, the channel design protruded 1 mm to imprint the channel onto the replica. The channel was designed to be 1.2 cm long and 3 mm wide, with a flat 1 mm profile in the center and inlet/outlet with cylinder shape of 1 mm radius each; the final 3D CAD that considers all these specifications is shown in (Figure 3a).

**Figure 3:**
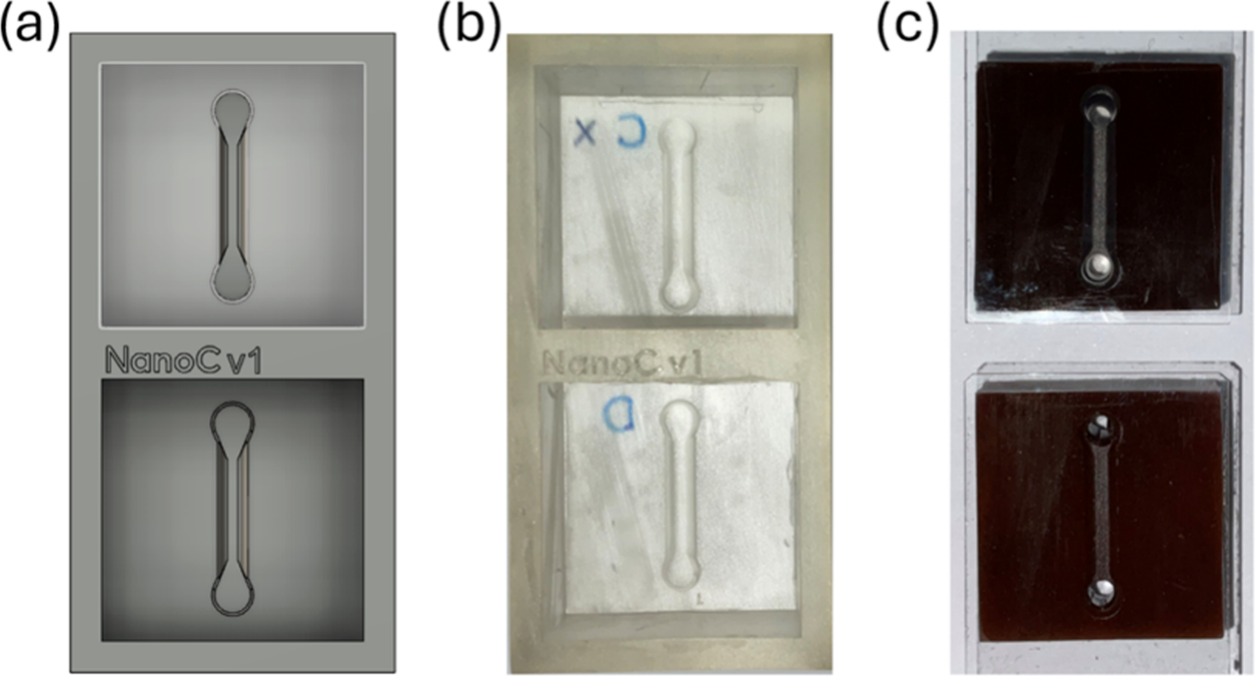
(a) Image of the 3D model of the mold used to replicate the MMDs. (b) Photograph of the mold, 3D printed with clear resin. (c) Photograph of final microfluidic devices after curation, punching, and sealing with a glass cover slide.

To parallelize the fabrication of devices, each mold had room for two devices and two molds, as the one shown in Figure 3b. SLA 3D printing was employed for that aim. Thus, we could make up to 4 devices that were later sealed by bonding them to a cover slide with an oxygen plasma machine. Examples of already sealed and punched MMDs can be seen in Figure 3c. After replication, the devices kept a square plan with a side width of 2.4 cm and a height of 8 mm.

Figure 3 summarizes all of the steps taken during the fabrication of the devices: mold design, materialization, replication, and preparation of the proposed microfluidic devices.

As discussed in the following sections, the final devices can reach and withstand a wide range of temperatures and fluxes.

### 3.3 Internal Channel Characterization and Roughness

The rugosity of the channel surface had an average roughness of (15 ± 1) μm; a volumetric 3D reconstruction was made to evaluate the channel quality, as shown in Figure 4. A closer perspective of the channel can be seen in Figure S7 in the Supporting Information, where the inner surface roughness of the channel is more palpable.

**Figure 4:**
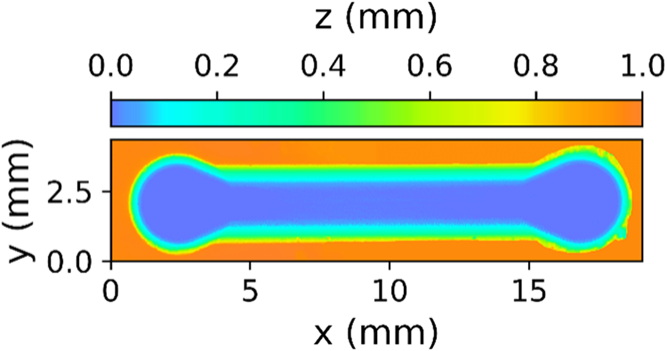
Processed height map of a 3D scan of the channel obtained by confocal microscopy. The rugosity inside the channel can be appreciated in Figure S7 of the Supporting Information.

The roughness of the surfaces inside and outside the channel was also photographed with SEM, where small clumps of nanoparticles were observed, as shown in Figure S2. The same test was performed on a control sample without IONPs, where the clusters were not appreciable, suggesting that, while IONPs are well integrated into the PDMS (change of color), there are still irregularities, which we will analyze in more depth in the next section.

Finally, the sample’s transmittance with IONPs was also analyzed in the NIR-UV spectrum with no significant transmittance windows, meaning that any experiment requiring observation of the inner channel would need an open geometry or a design with half a channel that can be pasted to a slide, like the one shown in this work.

### 3.4 Nanoparticle Distribution Analysis

To analyze the distribution of the IONPs in the whole volume, two devices with the same structure of microchannels were fabricated: one made of PDMS (control device) and a second one made of PDMS doped with the IONPs (magnetic microfluidic device). The high reflectivity of iron to X-rays makes it possible to detect the nanoparticle clusters as bright dots suspended in the PDMS. It allows us to determine the distribution of nanoparticles throughout the device.

Figure 5 depicts the nanoparticle cluster distribution over the whole volume of the magnetic microfluidic device. It can be observed that in the majority of the volume, nanoparticles are homogeneously distributed.

**Figure 5:**
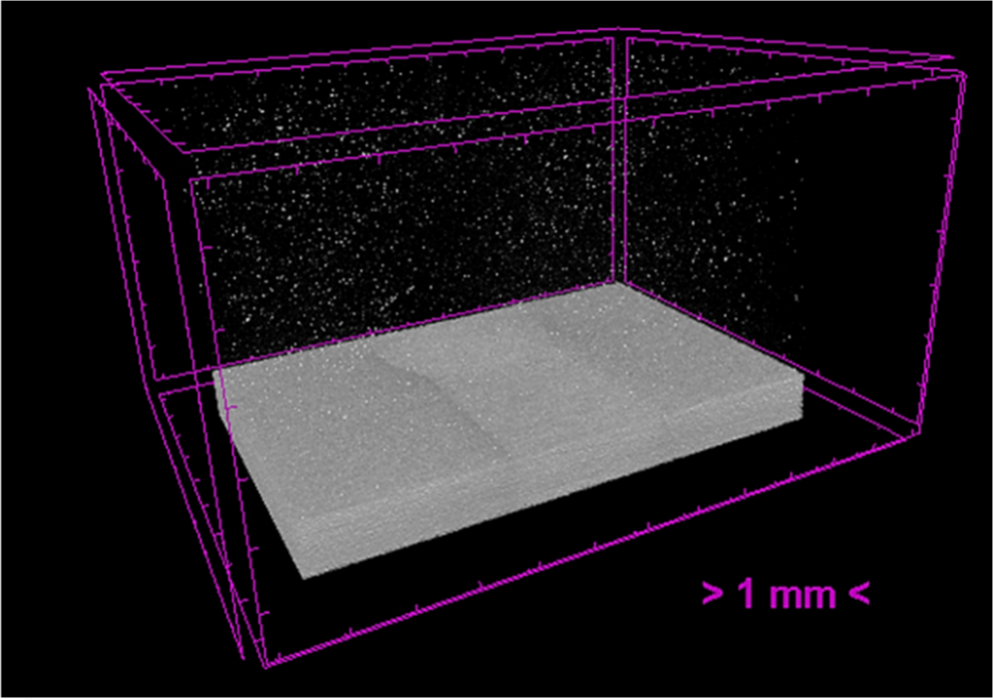
micro-CT image of a portion of the chip with IONPs. Bright spots correspond to the MNP clusters spreading throughout the chip.

The distribution of the clusters and the number of IONPs was analyzed and it is presented in Figure 6. The number of pixels of the micro-CT images that have a value higher than the maximum brightness (threshold) found in the sample with only PDMS (pixels brighter than PDMS are nanoparticle clusters) was determined. The pixels above this threshold (determined from the control made on PDMS) are due to the IONPs’ reflectance to the micro-CT X-rays, thus related to the total number of nanoparticles in the MMD. Figure 6c shows the IONPs distribution along the magnetic microfluidic device. The *X*-axis represents the height of the channel, starting at the cover slide, then moving to the channel and finally to the PDMS with nanoparticles. On the other hand, the *Y*-axis depicts the number of pixels exceeding the brightness threshold corresponding to the PDMS, which was determined from the brightness of the control PDMS device. The data are superimposed with a scheme of the device, where light gray represents the cover slide, black gray corresponds with the channel, and medium gray corresponds with the PDMS doped with magnetic particles. Figure 6a,b showcases the slices where a higher amount of IONP clusters can be seen. It reveals that the clusters of nanoparticles mainly tend to precipitate due to gravity during curation to the bottom of the device. This phenomenon can be seen at the two slices shown in Figure 6a,b, but also in Figure 6c, where proportionally to the rest of the volume, those two regions (marked by the two arrows in the enlarged plot) have a higher concentration of IONPs.

**Figure 6:**
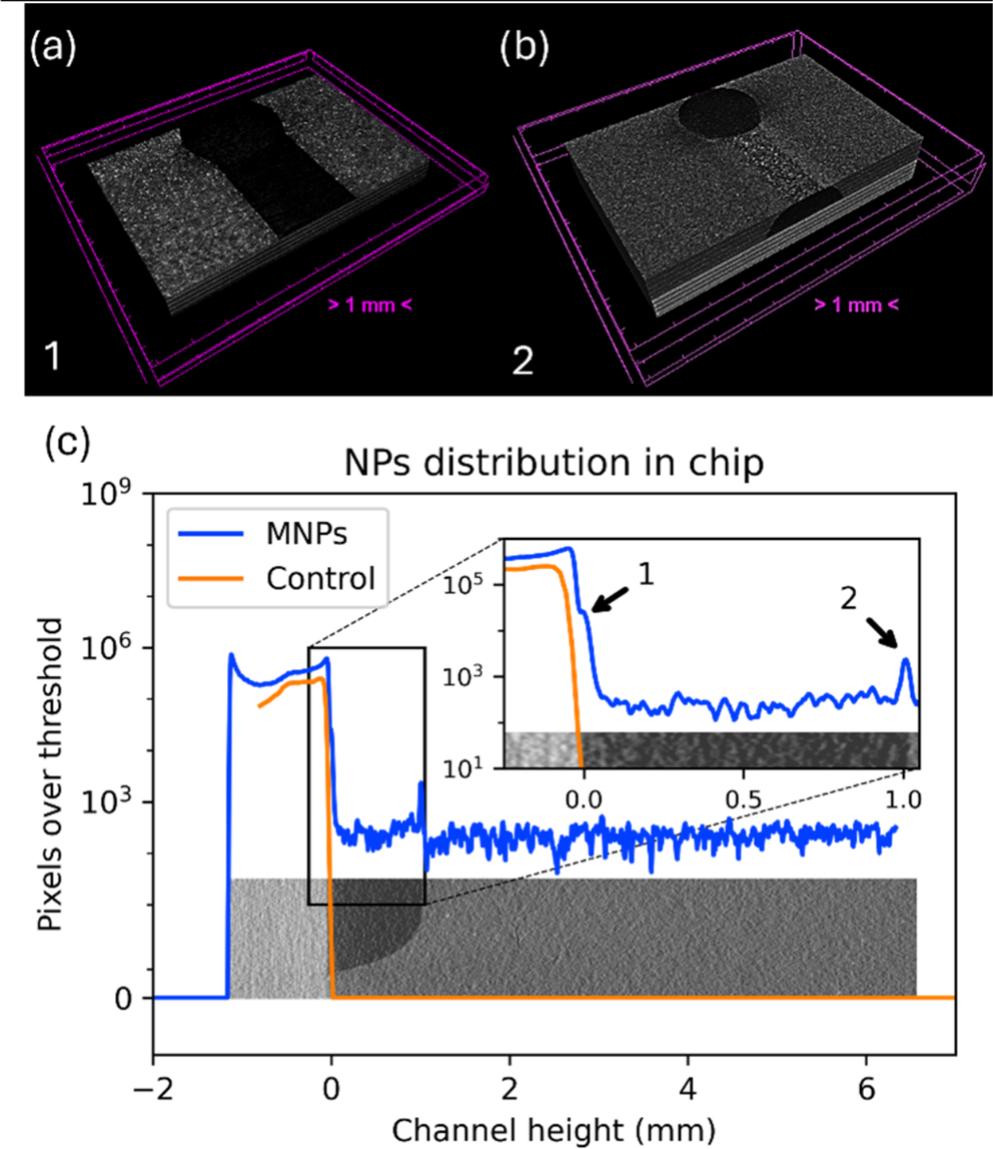
IONPs distribution of the chip. Slices of (a) bottom of the device and (b) top surface of the channel (c) showcase the distribution of the IONPs at the chip through the calculation of the pixels over the PDMS replica threshold superimposed with a scheme of the device. An enlarged view of the channel region is shown. Arrow 1 corresponds to the bottom of the channel, and arrow 2 corresponds to its top surface.

The analysis of IONPs shows that a homogeneous distribution of IONPs occurs. Moreover, a higher number of them are located at the top surface of the channel and at the bottom of the device (indicated in the enlarged view of Figure 6c with black arrows 1 and 2, respectively). Arrow 1 corresponds to the bottom of the channel (Figure 6a), whereas arrow 2 corresponds to its top surface (Figure 6b). We have also observed that the nanoparticle clusters also precipitated to the surface of the channel, which is evidenced by an increase of 19.4% of the mean brightness of the region between arrows 1 and 2, with respect to the volume above arrow 2 (region of the device above the channel). This phenomenon of precipitation may be attributed to the inability of PDMS to suspend nanoparticle clusters of more than a certain size or weight prior to the curation. It is important to note that even when precipitated, the particles are still sparsely distributed on the surface, making the majority of both surfaces to be covered in PDMS, supporting the approximation of simulating the chip with the thermal properties of PDMS.

### 3.5 Magnetic Hyperthermia Response

Remote thermal control of the MMDs was carried out through magnetic hyperthermia processes. For this purpose, the MMDs were introduced into the center of a cooled coil carrying ac electric current, which delivers a magnetic field of 0.16 kOe at a frequency of 361 kHz, while the temperature increase was recorded using an optical fiber. First, the magnetic hyperthermia response was studied under static conditions, i.e., without a flux inside the channel. Figure 7 shows the heating curves of the MMDs during 15 min of magnetic field application with different amounts of IONPs: (a) MMD_60_, (b) MMD_45_, (c) MMD_30_, and (d) MMD_15_. Table 2 compiles the maximum temperature increments obtained for samples MMD_60_, MMD_45_, MMD_30_, and MMD_15_, respectively, 58.20, 44.68, 38.90, and 33.47 °C, starting from room temperature. These results show that the temperature increases are related to the concentration of IONPs contained in the devices and that MMD_60_ and MMD_45_ provide thermal performance suitable for magnetic hyperthermia treatments.

**Figure 7:**
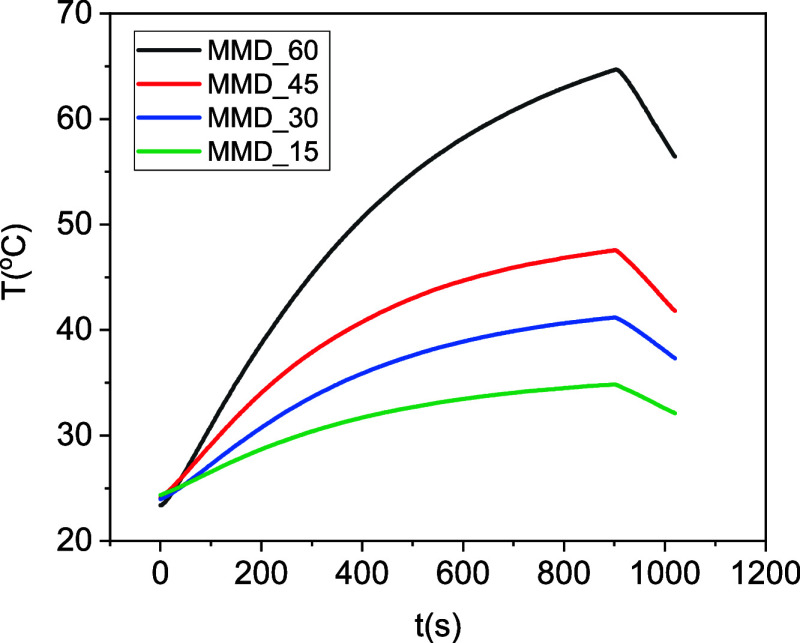
(a) Heating curves of MMD_60_ (black), MMD_45_ (red), MMD_30_ (blue), and MMD_15_ (green) samples developed under the application of a magnetic field of 0.16 kOe at a frequency of 361 kHz for 15 min under static conditions (without the presence of water flow).

**Table 2:** Temperature Differences Are Achieved for Each Device under 15 min of the Magnetic Field, where *m*_MNP_ is the Mass of Magnetic Nanoparticles in Each Device, *T_i_* is the starting Temperature, *T*_f_ Is the Temperature Reached after 15 min, and Δ*T* Is Their Difference^a^

sample	*m*_MNP_ (mg)	*T_i_* (°C)	*T*_f_ (°C)	Δ*T* (°C)
MMD_60_	60	23.38	58.20	34.82
MMD_45_	45	24.11	44.68	20.57
MMD_30_	30	23.98	38.90	14.92
MMD_15_	15	24.35	33.47	9.12

a Therefore, MMD_60_ was chosen as the prototype for studying the response of the system to different inner fluxes. The sampled peristaltic pump fluxes were (116 ± 3), (145 ± 4), (174 ± 4), (203 ± 5), (232 ± 6), and (261 ± 6) mm^3^ s^–1^.

### 3.6 Digital Twin

A digital twin was created as a virtual replica of the device and all of its surrounding elements. Using the hypothesis discussed in Section 2.7, the system was modeled as a heat source (the MMD) with several dissipation paths (air convection, heat conduction to other solids, and the inner water steam). The system was designed in Fusion360 as different pieces in contact. A rendering is depicted in Figure 8, where the different parts can be seen, and the surrounding air has been modeled as an invisible block. A more detailed view of the model can be seen in Figure S6, in the Supporting Information, where the cross section of the system and a transversal fluid section are presented. The digital twin was simulated in Star-CCM+ with the material properties of PDMS, as specified in Table 1. The distribution of nanoparticles obtained from the micro-CT images in Figure 6c is notoriously equally distributed in the regions with only doped PDMS, highlighting the idea of a homogeneous nanoparticle cluster distribution. This fact is in agreement with our results and, in particular, with our assumption that the device has the material properties of PDMS. The distribution of nanoparticles in clusters, isolated one from another, means most of the heat is conducted through PDMS, resulting in the device having approximately the properties of the PDMS itself.

**Figure 8:**
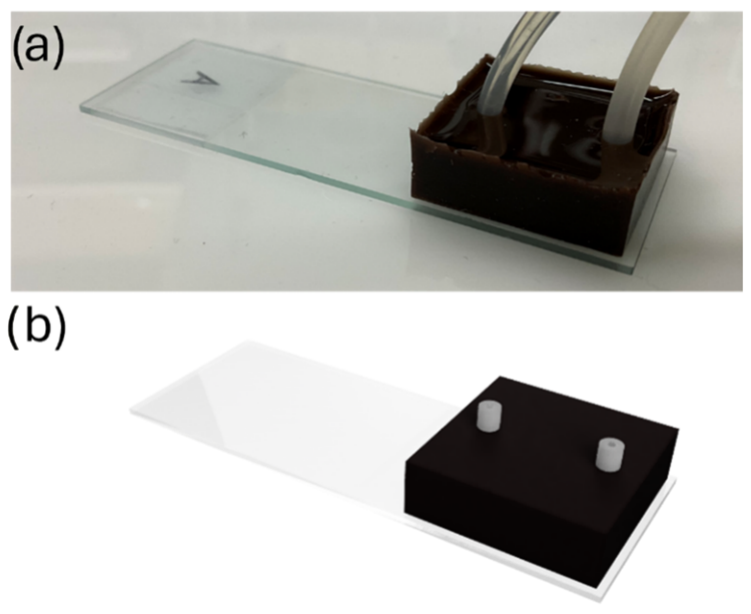
(a) Picture of the real MMD; (b) 3D rendering of the MMD’s digital twin. Each element has been modeled separately to be given different material properties and behaviors (air, glass, silicone, water, and the device itself).

All solids and liquids were meshed, and the interfaces between the different pieces allowed heat transmission. Since, in the experimental setup, the chip was inside a cylindrical tube, the flow was allowed only at the two corresponding interfaces of the modeled air around the device. Allowing the simulated air to dissipate energy through convection was fundamental to achieving a realistic simulation.

To assess the power of the device, we applied the hypothesis proposed in Section 2.7 to the fitted curves in Figure S1 in the Supporting Information. The differences in power fluxes observed in Figure S1b can be explained by slight differences in the alignment of the sample with the center of the magnetic field of the coil, which, due to its limited length, shows tiny variations in the intensity of the magnetic field the further one gets from the central axis. Therefore, the differences in the magnetic field seen by the corners of the MMD may explain these discrepancies. The fact that the heat production curves are not ordered by flux speed supports this hypothesis.

A model that gives a good idea of how a device will perform under different circumstances helps to optimize the work and material used to reach a specific goal. However, before a model can be used, it must undergo experimental calibration, which we show in the next section.

#### 3.6.1 Experimental Calibration

Flux experiments were made to test the effect of the device on the fluid temperature at the outlet. As stated before, the parameters fixed in each case were the power of the device and the inlet temperature, which were directly taken from the experimental data. The model was tested by comparing the temperatures obtained at the end of the channel at each instant in the experimental and simulated case, as shown in Figure 9, where Figure 9a,d,e,f have the best agreement between simulation and reality. Cases b and c are also in good agreement during a big part of the experiment but show discrepancies of up to 0.7 °C at 300 and 600 s, respectively.

**Figure 9:**
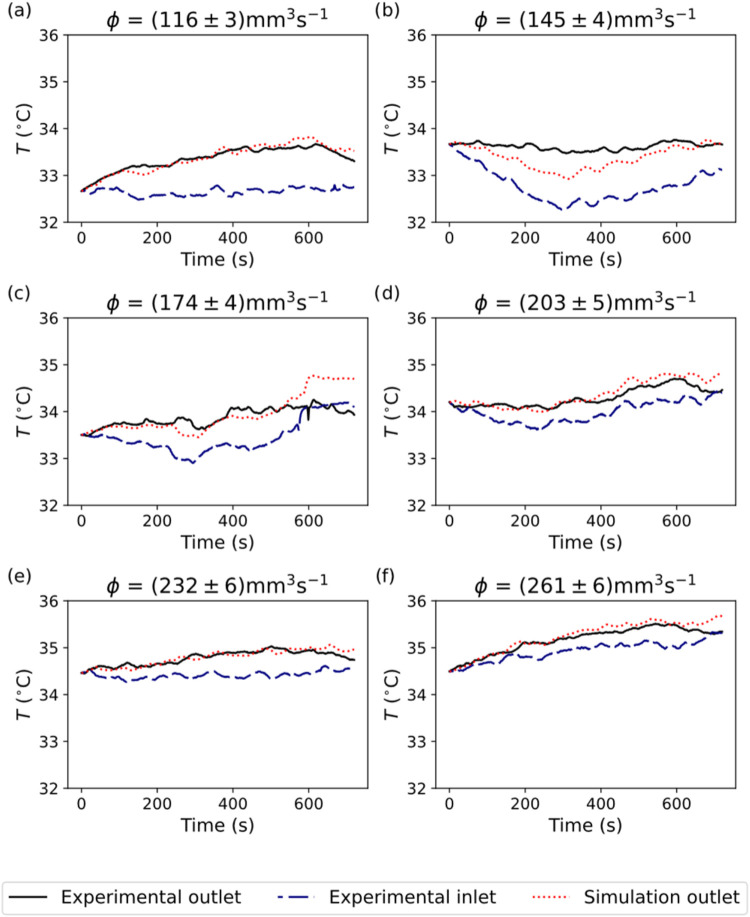
Comparison of fluxes (a) 116, (b) 145, (c) 174, (d) 203, (e) 232, and (f) 261 mm^3^s^–1^measured experimentally with their simulated counterparts. The initial temperature was the same for both cases (blue dashes), so the model could reproduce the liquid’s interaction with the device as desired. The simulated outlet temperatures (red dots) are compared to the experimental outlet temperature (black lines).

This behavior is probably due to the sudden change in the temperature of the liquid at the inlet, which can be easily fixed by keeping the liquid source at a more constant temperature, as in cases a, d, e, and f. Having two instances where the temperature drops and rises suddenly allowed us to determine the range of operation of our digital twin and validate its behavior. This limitation may be related to the thin and sparse layer on particles on the lower surface of the chip shown in the previous section. Modeling it would introduce a substantial complexity layer to the simulation that would translate in a small correction of the shown values, outside of the scope of this work, since the simulations still hold for the steady cases.

The temperatures of the device with an inner flux were also measured and simulated. The values are shown in the Supporting Information in Figure S5 and, compared with the corresponding experimental behavior at the different ϕ values. The first thing to notice is that the maximum temperature reached by the system after 600 s is ∼10 °C lower than the value measured without an inner flux in Figure 7. The new dissipation path allows the system to reach equilibrium at a much lower temperature. This can also be seen in the Supporting Information in Figure S1a, where the device without flux reaches a temperature difference of over 30 °C at equilibrium. This information is very valuable for the planification of future works.

Since the optic fiber was placed on the top surface of the MMD, the experimental temperature was compared with the average at the said surface and at the whole volume, the latter being in more agreement with the experiments, as shown in Figure S5 of the Supporting Information. Additional finetuning of the simulation parameters may be needed to achieve a better reproduction of the behavior of the chip. This should also be complemented by a deeper study on the properties of the MMD, which represents its own project and, thus, falls outside the scope of this work. Our model still works very well to predict temperature changes in liquid under stable conditions, easily achievable by using a thermostatic bath as a liquid source.

The results so far show the validity of our model, and thus, we can use it to analyze the internal distribution of the fluid temperatures within the device.

#### 3.6.2 Virtual Measurements

To exemplify one of the main advantages of our digital twin, we measured the temperature and the velocity of the liquid in a transversal section in the middle of the channel, as shown in Figure 10a,b, respectively. Due to the big size of the channel, we have reduced the flux to ϕ = 14.5 mm^3^ s^–1^, to make the temperature gradient in the fluid more evident.

**Figure 10:**
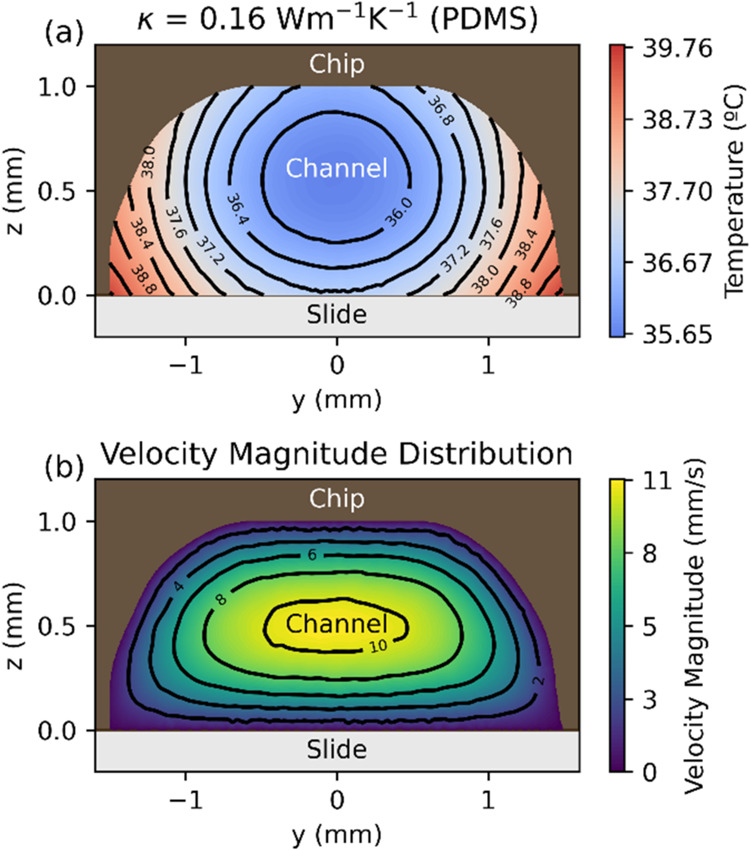
Cross section of the channel of the device perpendicular to the flow direction for a flux ϕ = 14.5 mm^3^ s^–1^. Panel (a) corresponds to the temperature map at the thermal conductivities associated with PDMS, whereas panel (b) depicts the associated velocity map. To help visualize, all plots have isolines, and the colormap has been fixed for all temperature maps. At *y* = 0, the velocity shows a near-parabolic profile. The temperature of the fluid at the inlet is 32.6 °C.

In Figure 10a, the minimum temperature is found closer to the chip than to the slide at first sight; this is counterintuitive since the element emitting energy is the chip, and the coldest region is usually the area where the flow is maximum, some tenths of millimeters lower, as shown in Figure 10b. Nevertheless, we hypothesize that having a chip with a thermal conductivity (κ) lower than water or the slide creates noticeable temperature gradients inside the chip. Heat flows from the chip to water and the slide at similar rates, but the slide receives more energy due to a bigger contact area. Heat then flows slightly faster to the water from the slide than from the chip, leading to the seemingly counterintuitive position of the minimum in the temperature profile. This hypothesis can be tested by increasing the thermal conductivity of the chip and showing how the position of the minimum gets closer to the slide as it approaches the more intuitive example of a highly conductive heating element. This is shown in Figure S9a,c,e, in the Supporting Information, where we sample three conductivities for the chip: PDMS, slide, and iron, confirming our previous hypothesis.

What we see is first that the higher the conductivity, the lower the difference between the maximum and minimum temperatures. We also see that the maximum temperature is much lower for the most conductive chip, perhaps because it is also the most dissipative of the three cases. And, once again, the minimum temperature is also displaced vertically toward the slide, confirming our previous hypothesis. Finally, the temperature isolines also seem to become more parallel to the interface as the conductivity increases.

It is also of interest to inspect how the different systems behave after the power of the device is turned off; this can be observed in Figure S9b,d,e, in the Supporting Information, where we see, as expected, how higher conductivities help dissipate faster and more uniformly the temperature of the water.

Since water is a better thermal conductor than PDMS, we also observe that it refrigerates the region of the chip directly on top of the channel, making the temperature isolines almost perpendicular to the flat observation surface. This property is not easily achievable with conventional heating systems (such as Figure S9e) and could, for example, help study cell migration mechanisms due to temperature gradients. This could be performed in a single chip, where the first half of the channel can be embedded in pure PDMS and the second half with doped PDMS. This means that, with a single experiment, it is possible to measure the response of cells that have been exposed at different known temperatures and also to check how the control cells behaved just by moving the chip horizontally without removing it from the reflection microscope or needing to change the focal point, thanks to the upper flat observation surface of the channel. The observations would be done through the slide, eliminating the problem of the chip being opaque.

The temperatures shown in Figure 10a and in Figure S9a,c,e were measured after exciting the system for 600 s at a constant power; if left turned on, the temperature would grow asymptotically. To control the temperature and keep it constant at a specific value, the chip could be turned on and off in a controlled manner to fix the temperature of the system. This application, again, exceeds the scope of this work, where we are setting the framework to develop the said tools. For a future project, we could add to the simulation a parameter, dependent on the temperature of the water at the outlet, that controls when to turn on or off the power of the chip, keeping the temperature map constant for prolonged periods of time if needed. This information could be acquired from the simulation and then used in the laboratory.

With these tests, we conclude that our device shows promising behaviors and applications not easily achievable through more conventional methods and opens up a new set of problems that Organs on a Chip can solve if implemented with this technology.

### 3.7 Potential Applications of the IONPs Integrated Microfluidic System

The integration of iron oxide-based magnetic NPs into microfluidic systems provides new functionalities to the OoC platforms, enabling remote localized heating through magnetic hyperthermia processes. The spatial and temporal control of temperature enhances the applicability and versatility of the OoCs, particularly as cellular evaluation systems in therapies involving magnetic hyperthermia. One of the most promising directions is controlled drug release, which can be actively induced by magnetic hyperthermia in nanoparticle-based delivery systems.^58,59^ By applying an external magnetic field, the induced hyperthermia triggers drug release specifically at the tumor site, thereby reducing the systemic side effects. This approach directly connects with the disease modeling paradigm in OoC platforms, where hyperthermia can be used as a tool to investigate cellular behavior under localized thermal stress. These studies are highly relevant for various tumor types, including glioblastomas, hepatocellular carcinomas, and breast cancer.^60–62^ Moreover, hyperthermia is emerging as a therapeutic modality in vascular processes. Specifically, restenosis, a pathological phenomenon characterized by the renarrowing (stenosis) of a previously treated blood vessel, appears to be susceptible to hyperthermia treatment. Locally induced thermal therapy in vascular environments could modulate smooth muscle cell proliferation and inflammation, thereby contributing to the prevention of neointimal hyperplasia. Previous studies have supported this concept, demonstrating the efficacy of thermal therapies in reducing restenosis.^63–65^ In the field of tissue engineering, magnetic hyperthermia has demonstrated the capability of providing precise and localized mechanical and thermal stimulation that promotes cell differentiation and proliferation. Magnetic nanoparticles enable the generation of mechanical forces and thermal signals within controlled microenvironments, activating key cellular pathways such as stretch-sensitive ion channels, which are critical for the formation of tissues such as cartilage and bone. Additionally, the anticancer properties conferred by nanoparticles to scaffolds further enhance their therapeutic potential.^66–68^

In summary, the proposed magnetic microfluidic platform demonstrates high adaptability and potential for a wide range of biomedical applications, spanning from personalized oncological therapies to complex tissue modeling and vascular disease treatment. This constitutes a significant potential advance toward the development of intelligent and thermally tunable OoC systems.

## 4 Conclusions

In this work, we successfully implemented magnetic microfluidic devices (MMDs) with biocompatible materials and created and validated a digital twin for them. The devices constructed are fully characterized, showing that iron oxide nanoparticles are homogeneously distributed throughout the device, albeit a subtle IONP accumulation is found on the boundaries of the channel. Temperature assays were performed with the chip, showcasing the functionality of magnetic hyperthermia. To go deeper into the study of the temperature distribution within the flow, a digital twin model was accomplished. Validation of the experimental assays was performed, showcasing that theoretical and experimental flow temperature distribution were alike.

Furthermore, cross-sectional analysis reveals a nonhomogeneous distribution of the temperature along the channel. At first sight, this phenomenon may seem problematic, but it can have its own applications like for the study of cell migration, as discussed in the previous section, which represents a very valuable behavior for applications in the fields of chemistry and medicine.

To summarize, this approach sheds light on the possibility of creating microfluidic devices with very fine temperature control. In our case, we designed the device with a very big channel to affect the output temperature as little as possible, but designing different sizes and geometries (with the help of the digital twin) might result in different temperature values and distributions. This tunability, along with the biocompatibility of the IONPs, turns them into good candidates to delve into biomedical applications related to oncology, drug delivery, and tissue engineering in different thermal stress scenarios. If needed, future studies could be performed with smaller channel diameters (implying higher fluid velocities and higher temperatures due to lower heat dissipation). Also, by designing them as half channels, we open the possibility for future studies of observing cell cultures through the cover slide by reflected light microscopy. The flat top surface of the channel would allow observations to be made without changing the focal plane.

The first steps have been accomplished on this proof of concept, although further studies will have to be performed in the future to display this potentiality in one of several novel applications.

## Supplementary Material



## Notes

Raw data from the experiments (micro-CT scans, experimental temperature measurements, etc.), as well as simulation parameters, have been uploaded to an open repository hosted in the following link: Software - Photonics4life (PDF)

## Notes

None of the authors have different addresses than those shown in the affiliations line

## Abbreviations

OoC: organ on a chip
NPs: nanoparticles
MNPs: magnetic nanoparticles
IONPs: iron oxide nanoparticles
PDMS: polydimethylsiloxane
MMD: magnetic microfluidic device
XRD: X-ray diffraction
fwhm: full width at half-máximum
TEM: transmission electron microscopy
ATR: attenuated total reflectance
SI: Supporting Information
SEM: scanning electron microscopy
OLA: oleylamine
NIR-UV: near infrared-ultraviolet
FeSO4·7H2O, 99%: iron­(II) sulfate heptahydrate
FeCl3·6H2O, 99%: iron­(III) chlorhide hexahydrate
NH4OH, 28%: ammonium hydroxide solution
C6H12, 99%: cyclohexane
