# Supplementary material for: Thermoregulable Magnetic Microfluidic Devices by Magnetic Hyperthermia from Iron Oxide Nanoparticles
Source: ACS Appl Nano Mater. 2025 Jul 11;8(29):14505–18. doi: 10.1021/acsanm.5c01562 (PMC12308749; doi:10.1021/acsanm.5c01562)
Supplement: Supplementary file 1 [file an5c01562_si_001.pdf]

Supporting Information

Thermoregulable Magnetic Microfluidic Devices by Magnetic Hyperthermia from Iron Oxide Nanoparticles

Santiago, Parames-Estevez[a,b] , Pelayo, García Acevedo[c], Yago, Radziunas-Salinas[d], Yolanda, Piñeiro[c], José, Rivas [c], Maria Teresa, Flores-Arias[*d], Alberto, P. Munuzuri [*a,b]

[a]Group of Non-Linear Physics. Campus Sur. University of Santiago de Compostela, Spain.

[b]Galician Center for Mathematical Research and Technology (CITMAga), Santiago de Compostela, Spain.

[c]Nanotechnology and Magnetism Lab — NANOMAG ; Materials Institute - iMATUS ; Health Research Institute – IDIS; Department of Applied Physics; Universidade de Santiago de Compostela.; E-15782 Santiago de Compostela; Spain.

[d]Photonics4Life Research Group, Applied Physics Department, Faculty of Physics and Materials Institute - iMATUS , 15782 Universidade de Santiago de Compostela, Campus Vida, Santiago de Compostela, Spain

As explained in the main document, the temperature for the device at each experiment was monitored over time, and with it, the heat flux observed by the magnetic microfluidic device (MMD) was calculated. This has been represented in Figure S1, where all the studied cases have been compared.

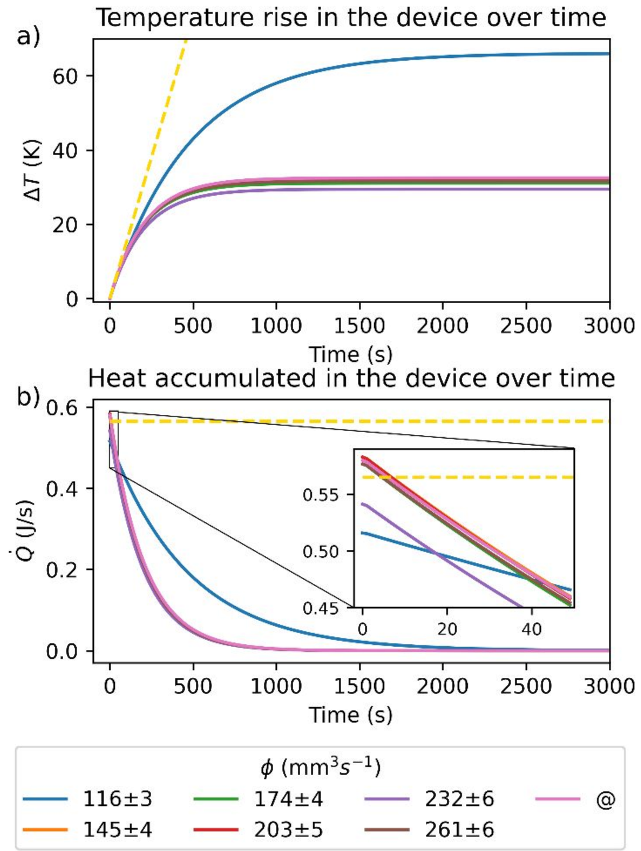

Figure S1. Increase in temperature a) and heat flux b) curves over time for the device at each experiment. In a golden discontinuous line, the hypothetical behaviour of an adiabatic device (@) of power 0.57 W. The start of the curves in b) has been zoomed to a subplot to show how the different lines intersect. Experimental temperature curves in a) were fitted to Equation 3 and extrapolated until equilibrium. The $\dot{Q}$ associated with each one was calculated with the time derivative of each fit in b).

To verify how homogeneous is the mixture of PDMS and magnetic nanoparticles (MNPs), a spectral electron microscopy image was taken for a device with MNPs (Figure S2) and without (Figure S3). Small clusters can be seen in the device with MNPs. Since they do not appear in the control image, we conclude they are clusters of MNPs. Even if the device is sufficiently mixed to work, microscopically, the particles aggregate, which may

explain why the digital twin works better when the properties of PDMS are assumed. The majority of the volume is PDMS, and the small clusters of MNPs heat the whole chip.

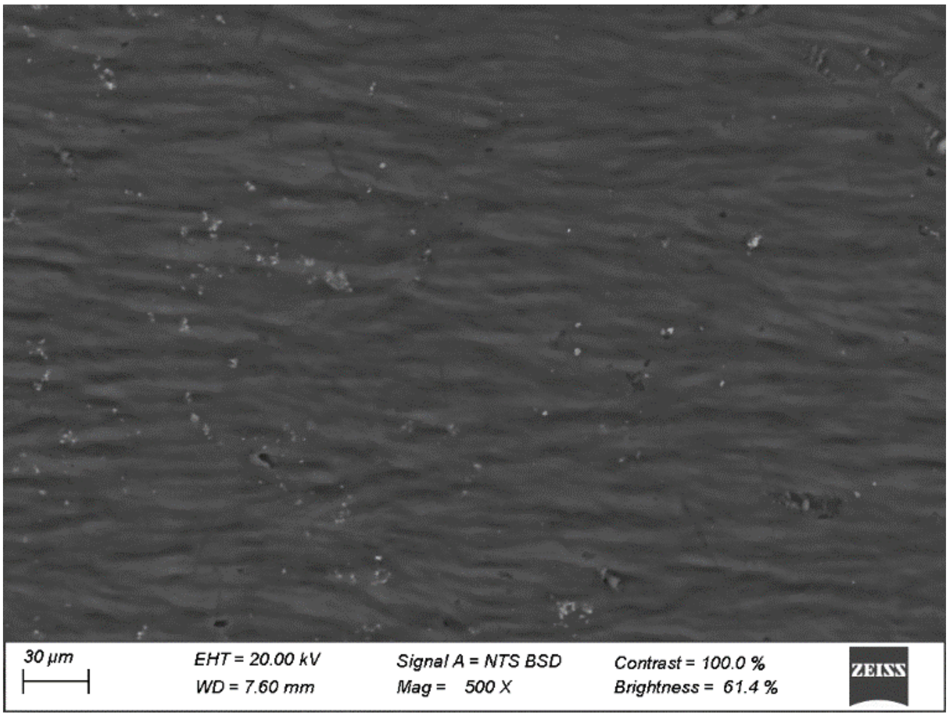

Figure S2. SEM image of the device with MNPs integrated. Small and bright clusters of dots can be seen on the surface.

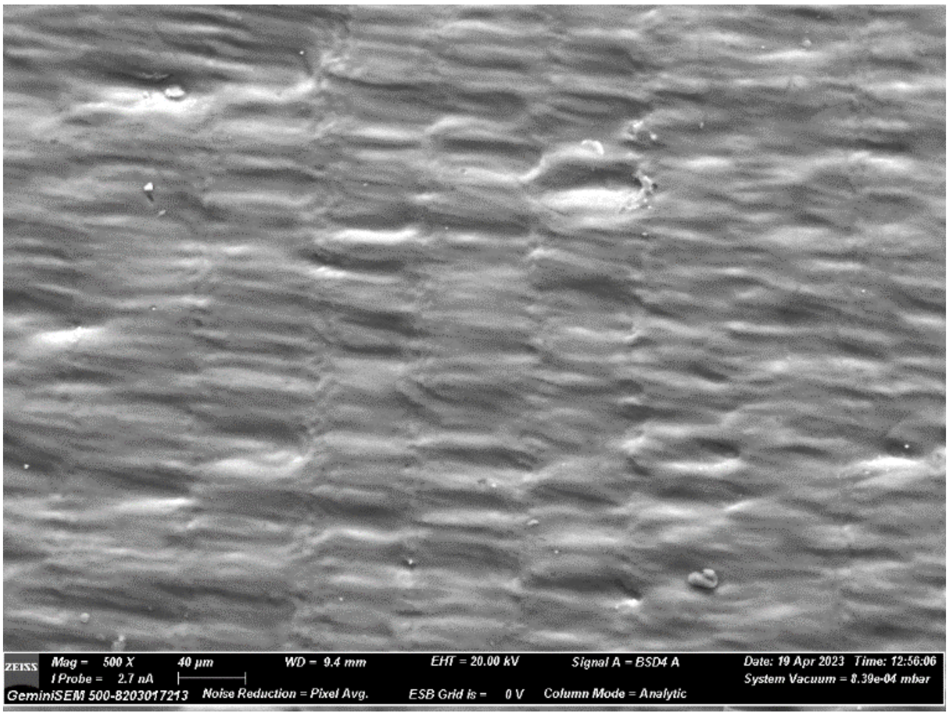

Figure S3. SEM image of a control device without MNPs. The small clusters of bright dots observed in Figure S2 were not observed.

In Figure S4 the MNPs' properties are discussed and used to characterize their composition. This information confirms the cubic spinel structure of the nanoparticles and also the presence of Fe-O bonds. This is discussed in more depth in the Results section of the primary document.

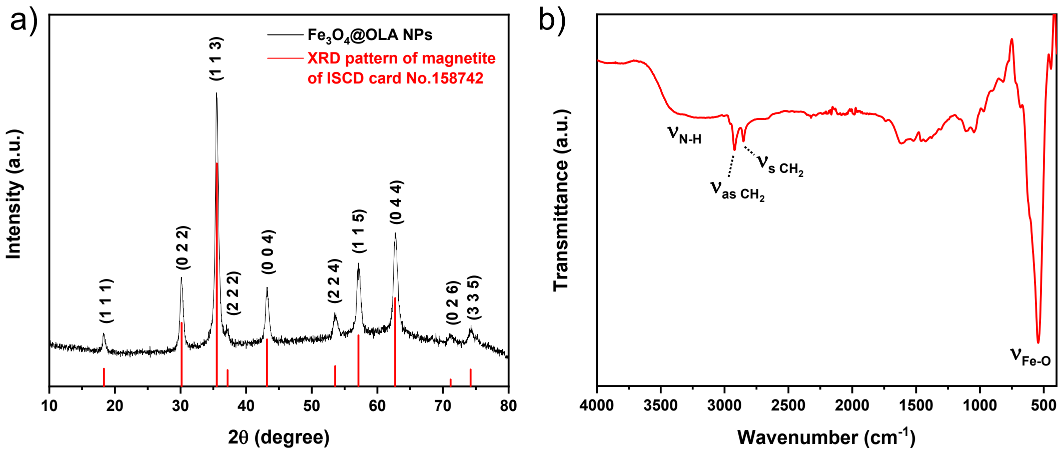

Figure S4. a) XRD pattern of magnetite and the synthesized magnetic nanoparticles. b) FT-IR spectrum of the prepared Fe_3O_4@OLA MNPs.

The behavior of the simulated temperature of the digital twin has been compared to the experimental values in Figure S5, where the average temperature of the whole volume is in better agreement with the average only at the surface, but there is still a substantial difference, probably due to some of the assumptions made, like making the digital twin have the properties of PDMS, this simplification is still enough to describe the temperatures in the liquid, as shown in the main document.

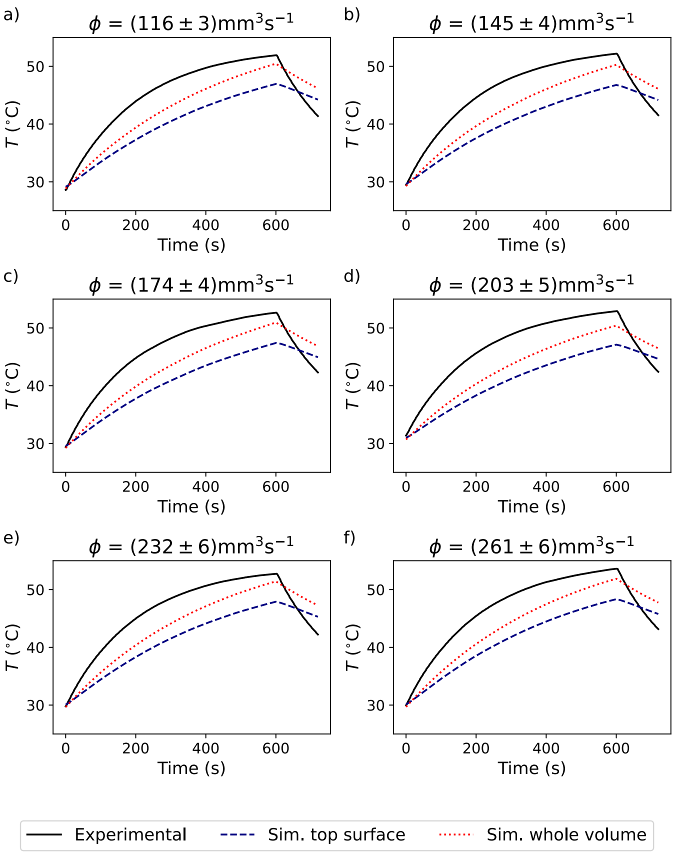

Figure S5. Comparison of MMD experimental temperature (black line) measurements with the averages in the simulation at the whole volume (red dots) and at the top surface (blue dashes), where the sensor was placed experimentally.

In Figure S6 a cross-section of the digital twin is shown to illustrate the configuration of its internal components; the air was also modeled as an invisible block around the device. In Figure S7 this image shows an inner perspective of the channel scanned with a confocal microscope, which shows the surface's rugosity.

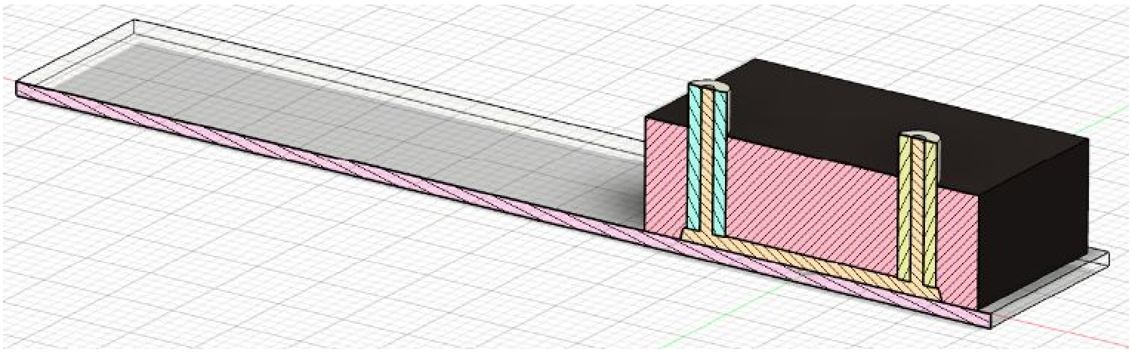

Figure S6. Cross section of the digital twin, where the fluid can be differentiated from the MMD, the silicon tubes, and the slide.

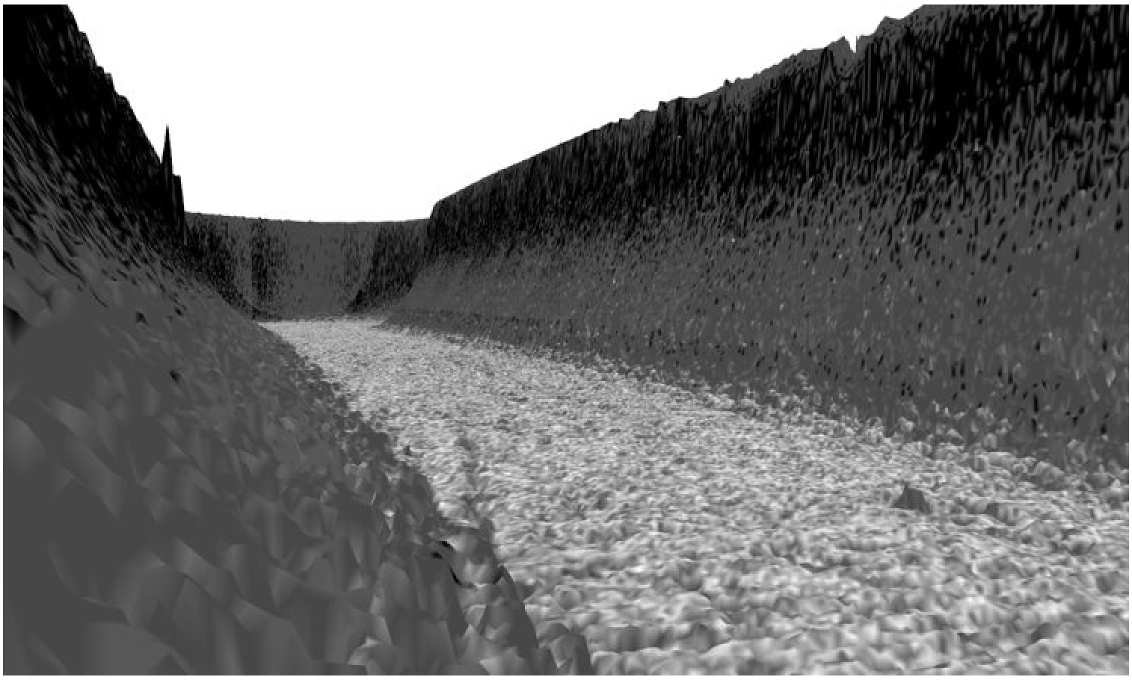

Figure S7. Confocal images: closer perspective of the channel from the inside. The channel is 1 mm tall, and the ridges are 15 μm in average. The flat surface is 1 mm wide.

In Figure S8, a section of the MMD scanned with micro-CT is shown. In each image, a different brightness threshold is chosen to show pixels above it. In Figure S8a, all of them are shown, in b the brightest regions of the PDMS along with the MNP clusters, and finally, in c, only the MNPs. In all three images, the bright slab at the bottom represents the glass slide, which also reflects the X-rays.

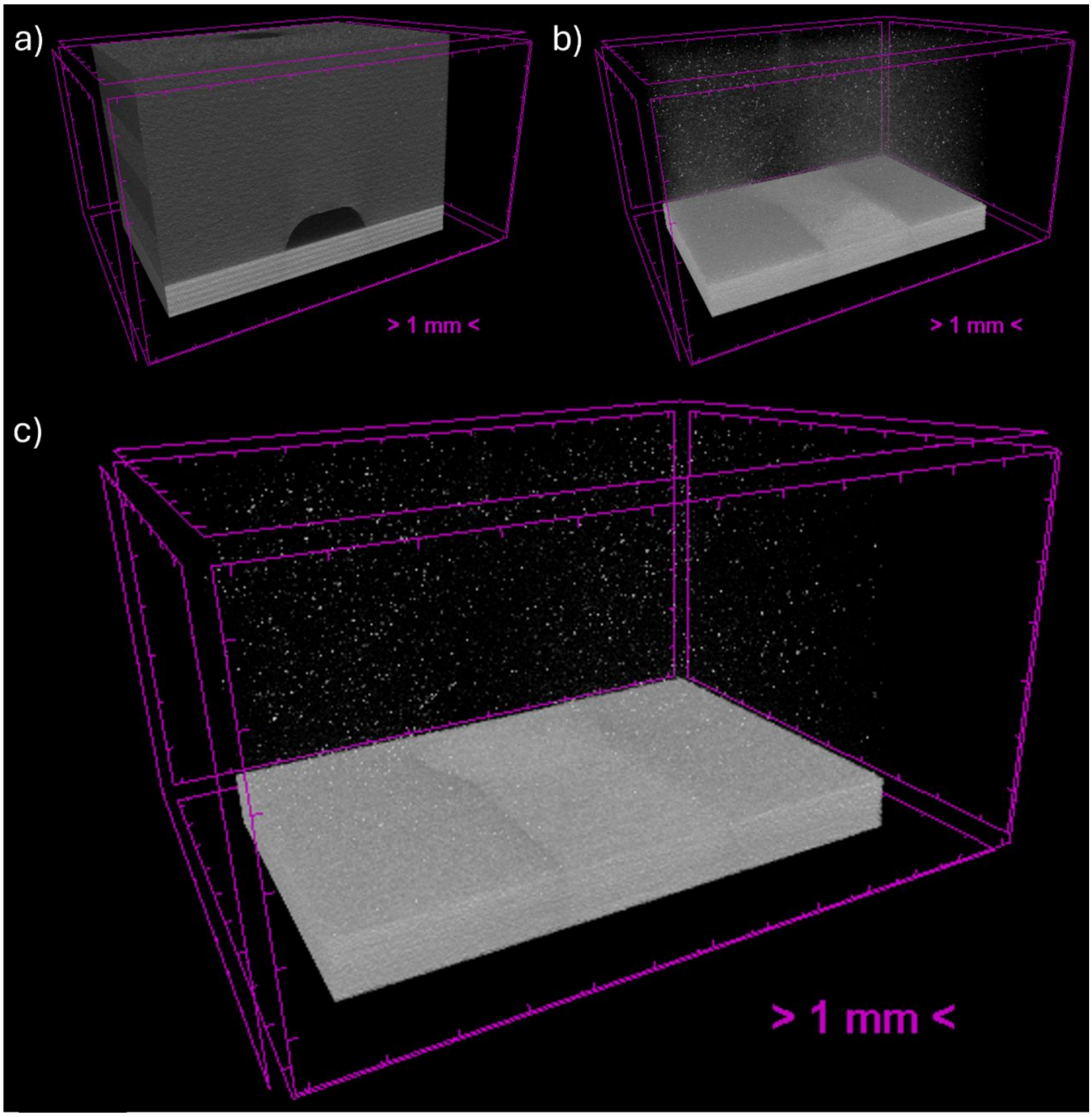

Figure S8. Micro-CT images: a scan of the doped device showing pixels with brightnesses over different thresholds. a) shows everything, b) the brightest parts of the PDMS and the MNP clusters, and c) only the MNP clusters.

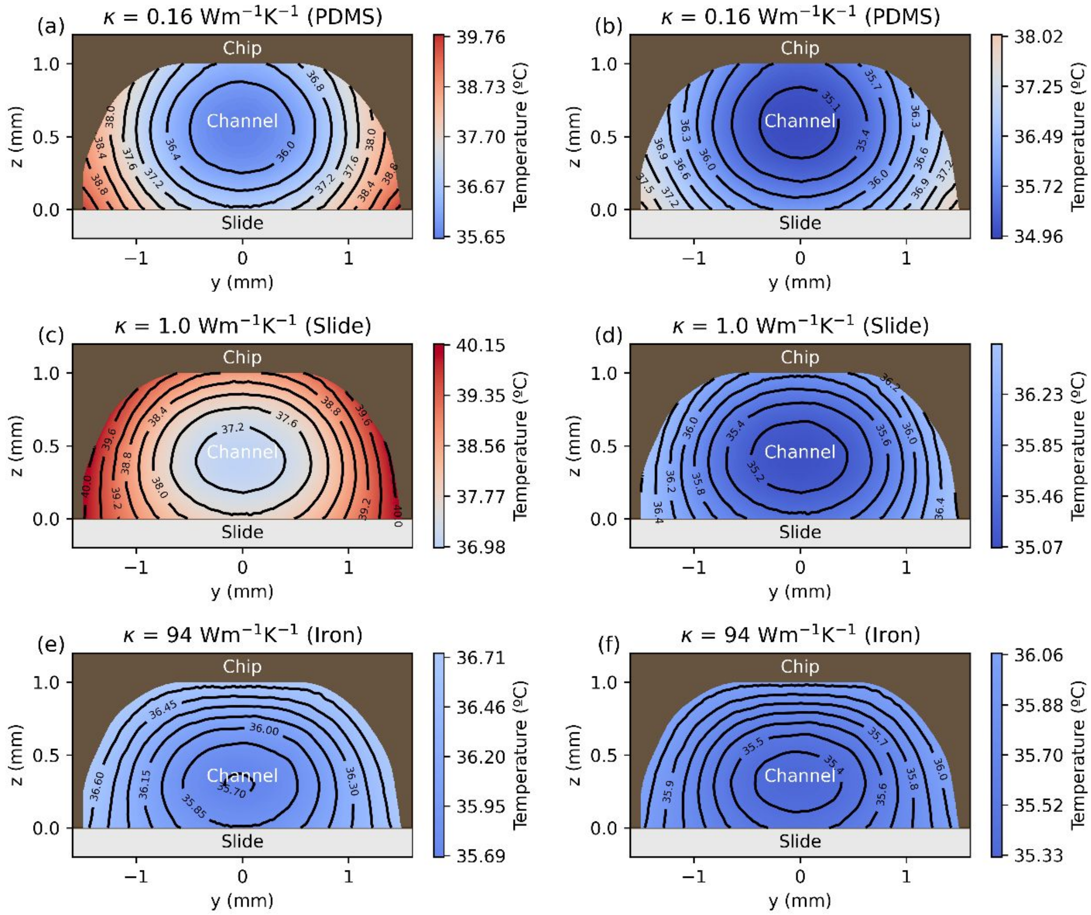

Figure S9. Temperature maps for a transversal section of the channel at its middle point. The power emitted by the chip is turned off at 600s, a, c, and e have been taken exactly at that instant, while b, d and f, 120 s later, allowing the heat to dissipate. This Figure shows the differences in behaviour when the thermal conductivity (κ) of the chip is set to that of PDMS (a, b), the slide (c, d) or Iron (e, f).

Finally, in Figure S9, the temperature in the fluid is compared at three different scenarios for two time instants (temperatures after 600s with the chip turned on, and 120 s later, after turning it off), where the thermal conductivity (κ) of the chip is varied. This variation was made to prove our hypothesis on why the minimum temperature is closer to the chip than to the slide in the digital twin of our chip (Figure S9b), as discussed in the main document. The more conductive the chip is, the faster and with less spatial variation, the temperature is dissipated.
